# Supplementary material for: Adversarially-Regularized Mixed Effects Deep Learning (ARMED) Models Improve Interpretability, Performance, and Generalization on Clustered (non-iid) Data
Source: IEEE Trans Pattern Anal Mach Intell. Author manuscript; Available in PMC 2023 Nov 14. (PMC10644386; doi:10.1109/TPAMI.2023.3234291)
Supplement: supp1-3234291 [file NIHMS1906724-supplement-supp1-3234291.pdf]

Adversarially-regularized mixed effects deep learning (ARMED) models improve interpretability, performance, and generalization on clustered (non-*iid*) data: **Supplemental Materials**

Kevin P. Nguyen, Alex H. Treacher, & Albert A. Montillo

Contents

1 Supplemental tables **1**

2 Supplemental figures **2**

3 Supplemental methods **10**

3.1 Specific mixed effects model architectures 10

3.1.1 Dense feedforward neural network (DFNN) 10

3.1.2 Convolutional neural network (CNN) 10

3.1.3 Autoencoder-classifier (AEC) 11

3.2 Spiral classification simulations 12

3.3 Alzheimer's Disease and mild cognitive impairment clincal and neuroimaging datasets 12

3.3.1 MCI conversion prediction dataset 12

3.3.2 AD diagnosis dataset 13

3.4 Melanoma live-cell image dataset 14

1 Supplemental tables

Table S1: Characteristics and MRI acquisition parameters for ADNI2 and ADNI3 study sites included in Alzheimer's Disease diagnosis training dataset.

Site ID	Subjects	Images	% AD	Manufacturer	Model	Flip angle	TE (ms)	TI (ms)	Resolution (mm)
52	8	14	85.7	General Electric	Discovery MR750	11°	3	400	$1.2 \times 1.1 \times 1.1$
5	7	16	81.3	General Electric	Discovery MR750	11°	3	400	$1.2 \times 1.0 \times 1.0$
126	12	26	76.9	General Electric	Discovery MR750	11°	3	400	$1.2 \times 1.0 \times 1.0$
57	6	9	55.6	General Electric	Discovery MR750	11°	3	400	$1.2 \times 1.0 \times 1.0$
16	21	56	50.0	General Electric	Signa HDxt	11°	3	400	$1.2 \times 1.0 \times 1.0$
2	24	80	11.3	Philips	Intera	9°	3	900	$1.2 \times 1.1 \times 1.1$
100	15	28	7.1	Philips	Achieva	9°	3	900	$1.0 \times 1.0 \times 1.0$
73	16	56	8.9	Siemens	TrioTim	9°	3	900	$1.2 \times 1.1 \times 1.1$
41	27	79	3.8	Siemens	TrioTim	9°	3	900	$1.2 \times 1.1 \times 1.1$
22	9	30	3.3	Siemens	TrioTim	9°	3	900	$1.2 \times 1.1 \times 1.1$
941	29	66	1.5	Siemens	Prisma Fit	9°	3	900	$1.0 \times 1.1 \times 1.1$
20	10	22	0.0	Siemens	Prisma Fit	9°	3	900	$1.0 \times 1.0 \times 1.0$

AD: Alzheimer's Disease; TE: echo time; TI: inversion time

2 Supplemental figures

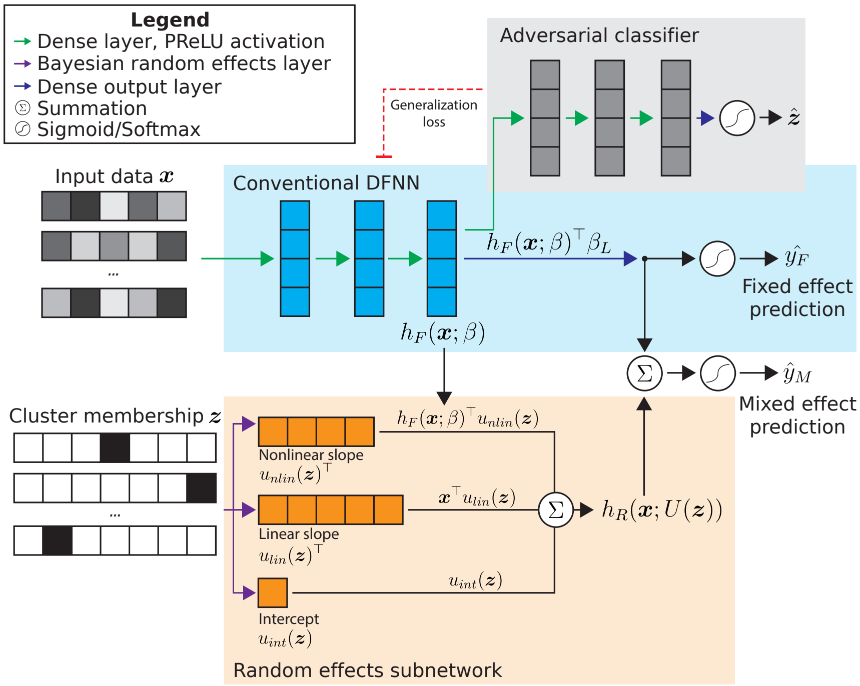

Figure S1: ARMED dense feedforward neural network (ARMED-DFNN). A conventional DFNN (blue area) applies a nonlinear transformation to the input data sample $\boldsymbol{x}$, yielding $h_F(\boldsymbol{x}, \beta)$ as the output from the penultimate dense layer. The final dense layer with weights β_L then produces a fixed effects-only prediction $\hat{y}_F$. The fixed effects subnetwork consists of this conventional DFNN and an adversarial classifier (gray area) which, through the generalization loss, penalizes the DFNN for learning features that are predictive of the sample's cluster $\hat{\boldsymbol{z}}$. The random effects subnetwork (orange area) learns normally-distributed weights $U(\boldsymbol{z}) \sim N(0, \Sigma)$ dependent on cluster $\boldsymbol{z}$. These include nonlinear slopes of $h_F(\boldsymbol{x}, \beta)$, linear slopes of $\boldsymbol{x}$, and/or intercepts. The combination (e.g. sum) of the fixed and random effects subnetwork outputs produces the mixed effects-based prediction $\hat{y}_M$.

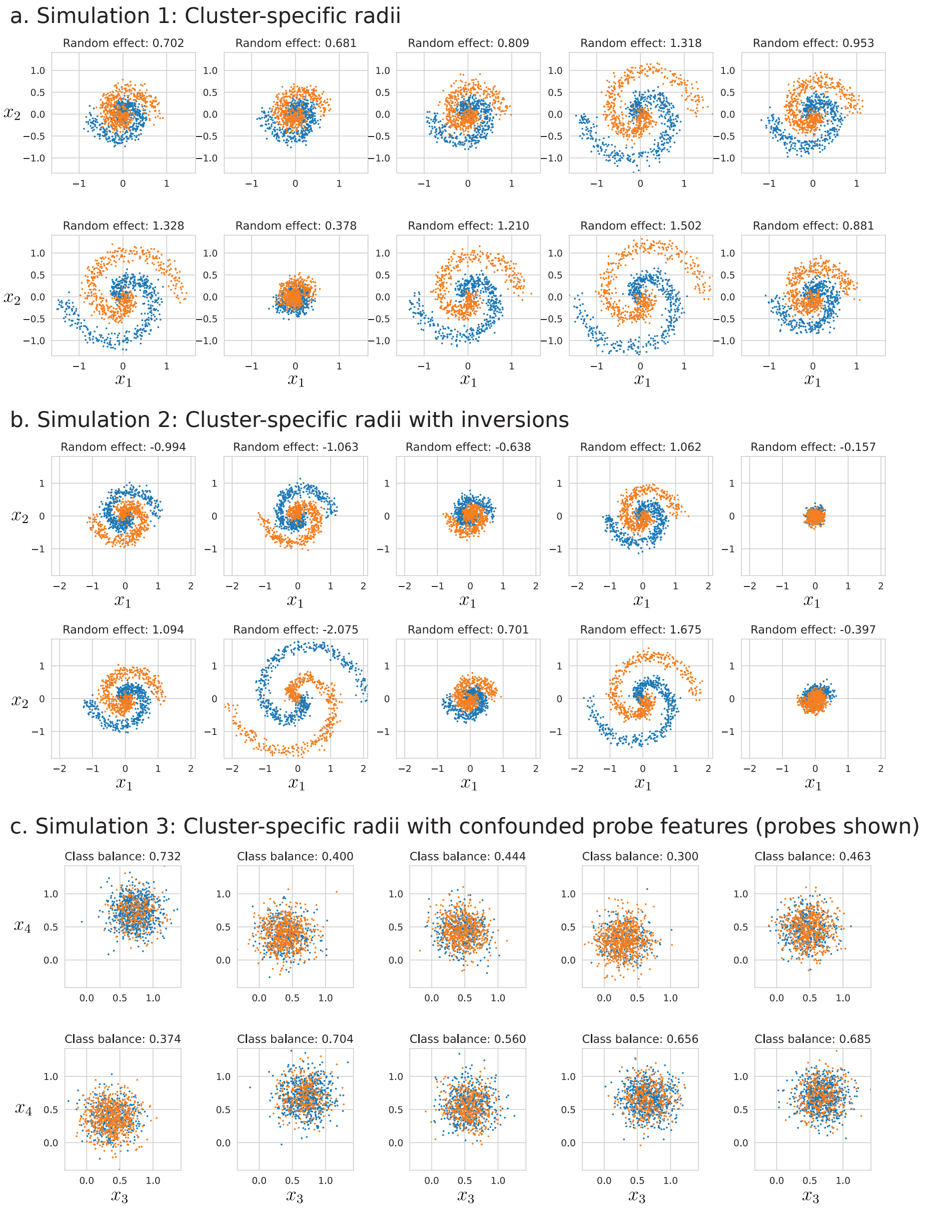

Figure S2: Spiral simulation datasets. Each simulated point has two features, x_1 and x_2, and the prediction target is whether it belongs to the blue or orange spiral. a) In simulation 1, 10 clusters were generated with cluster-specific spiral radii sampled from a normal distribution with $\mu = 1$ and $\sigma = 0.3$. b) In simulation 2, the cluster-specific radii were sampled from a normal distribution with $\mu = 0$ and $\sigma = 1$ such that spiral labels were inverted when the radius < 0. c) Simulation 3 was generated using the same parameters as simulation 1. However, two confounded probe features x_3 and x_4 were added, shown here, which were correlated with the ratio of blue-to-orange points but unassociated with the underlying spiral function.

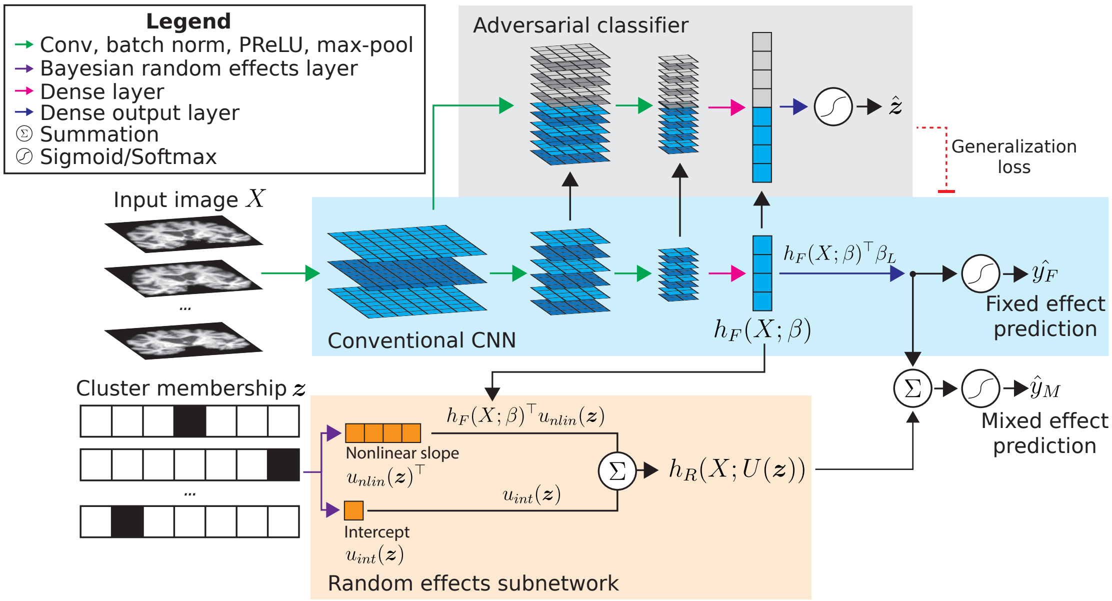

Figure S3: ARMED convolutional neural network (ARMED-CNN). For the conventional CNN (blue area), the penultimate dense layer produces a latent representation $h_F(X, \beta)$ of the data X. A final dense layer with weights β_L produces the classification prediction $\hat{y}_F$. To create the fixed effects subnetwork, an adversarial classifier (gray area) is added that learns to predict the sample's cluster $\hat{z}$ from the outputs of the layers of the CNN. The generalization loss penalizes the CNN for learning features that allow the adversarial classifier to predict cluster $\hat{z}$. The random effects subnetwork (orange area) learns normally-distributed weights $U(Z) \sim N(0, \Sigma)$ dependent on cluster $\hat{z}$, including scalars (nonlinear slopes) applied to $h_F(X, \beta)$ and a bias. These are combined with the fixed effects subnetwork output to yield the mixed effects-based prediction $\hat{y}_M$. Note: for illustration, not all CNN layers are shown.

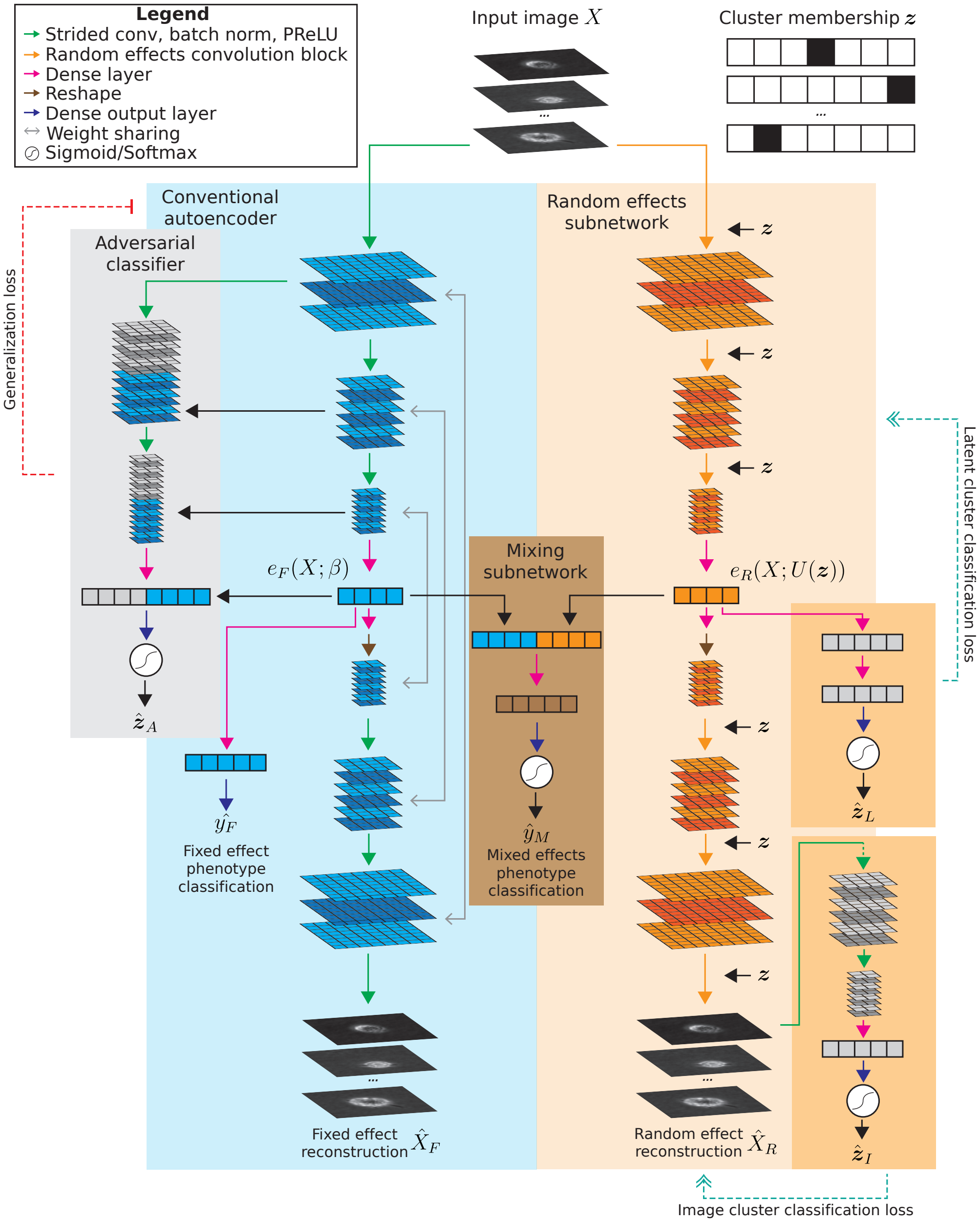

Figure S4: ARMED autoencoder-classifier (ARMED-AEC). A conventional autoencoder-classifier (blue area) contains an encoder that compresses the input image X into a latent representation $e_F(X;\beta)$, an auxiliary classifier that predicts the phenotype label $\hat{y}_F$, and a decoder that reconstructs the image $\hat{X}_F$. The fixed effects subnetwork combines this conventional model with an adversarial classifier (gray area), which penalizes the encoder through the generalization loss for learning features predictive of cluster membership $\hat{\boldsymbol{z}}_A$. The decoder shares weights with the encoder and so receives the same adversarial guidance. Meanwhile, the random effects subnetwork (orange areas) is a parallel autoencoder using random effects convolution blocks with cluster-dependent weights (Fig. S5). Its encoder compresses the image into a latent representation $e_R(X;U(\boldsymbol{z}))$ containing information predictive of cluster $\hat{\boldsymbol{z}}_L$. Its decoder then reconstructs an image $\hat{X}_R$ that is also contains information predictive of cluster $\hat{\boldsymbol{z}}_I$. A learned combination of the two latent representations yields the mixed effects-based phenotype prediction $\hat{y}_M$. Note: for illustration, not all autoencoder layers are shown.

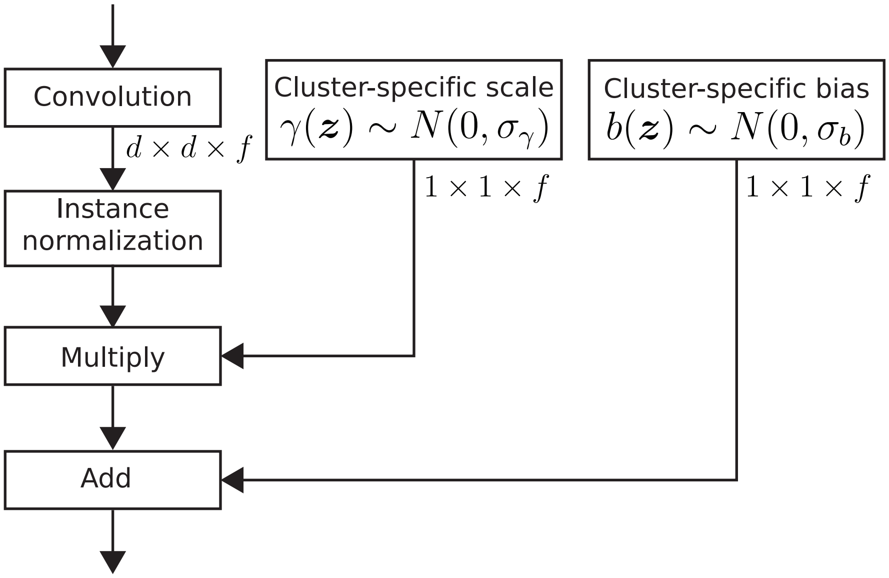

Figure S5: Random effects convolution (RECON) block used in the proposed mixed effects autoencoder-classifier (ARMED-AEC). A convolutional layer with f filters yields a $d \times d \times f$ output tensor. Instance normalization is applied, centering and rescaling values to zero mean and unit variance. A cluster-specific scale $\gamma(\boldsymbol{z})$ and bias $b(\boldsymbol{z})$ are then applied to each of the f feature maps. These cluster-specific scales and biases are regularized to follow normal distributions with learned variances σ_γ and σ_b, respectively.

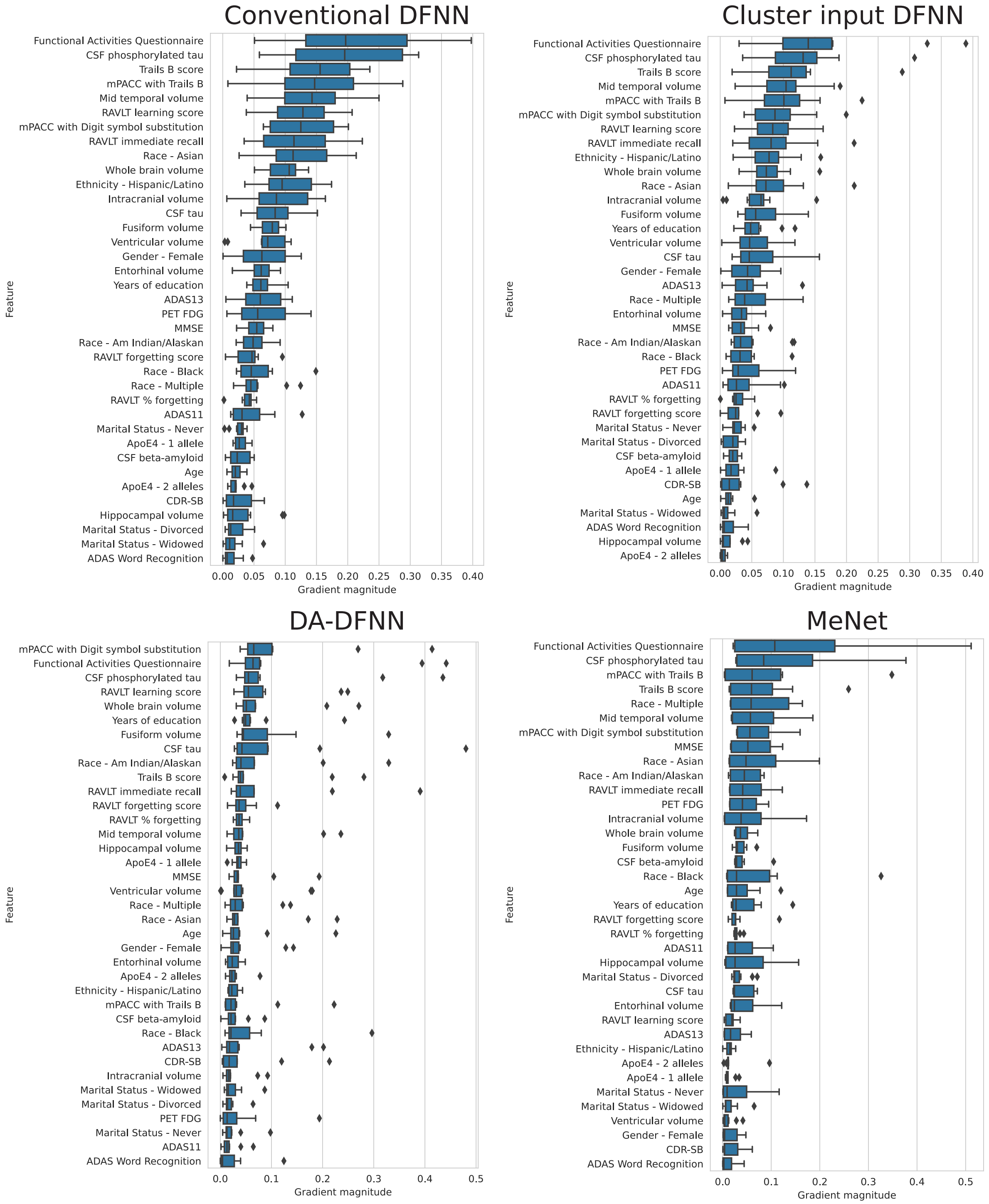

Figure S6: Feature importance in classification of stable vs. progressive mild cognitive impairment with various DFNN models. Features are ranked by descending median feature importance (gradient magnitude) across 10 cross-validation folds.

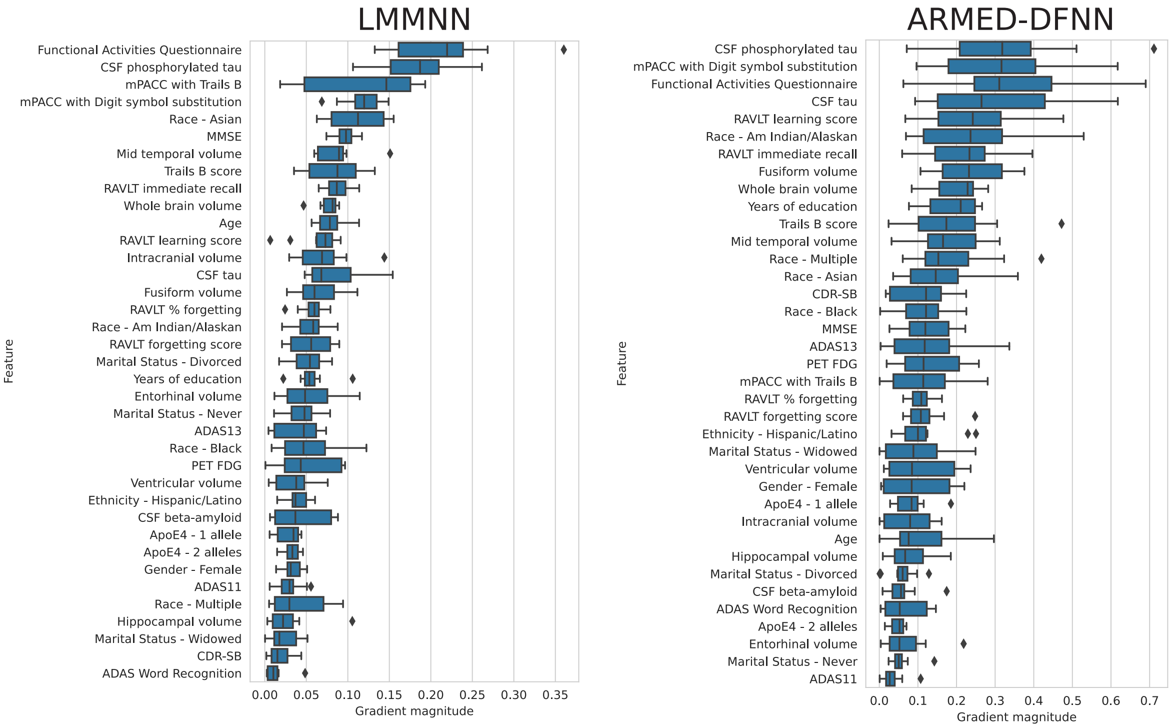

Figure S6: Feature importance in the classification of stable vs. progressive mild cognitive impairment with various DFNN models (continued).

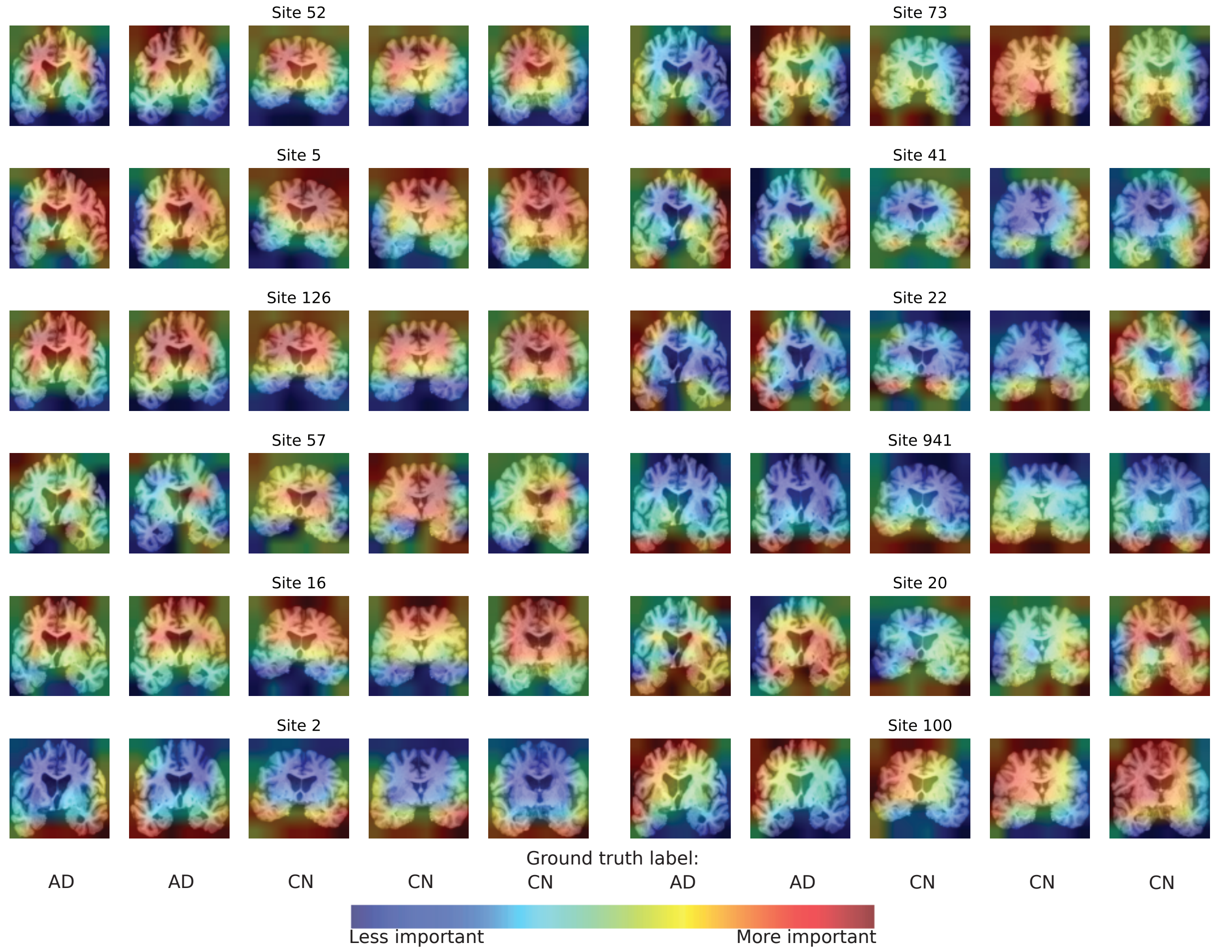

Figure S7: Site-specific random effects involved in Alzheimer's Disease diagnosis, as learned by the ARMED-CNN. For the same representative 5 images, we computed two Grad-CAM visualizations for each site j: G_F using the fixed effects subnetwork only and $G_M(j)$ using the full model applying the specific random effects for site j. Shown here are the difference images $(G_F - G_M(j))$ which reveal the contribution of the random effects subnetwork in predicting diagnosis. Colormaps are scaled per image. Ground truth labels, Alzheimer's Disease (AD) or cognitively normal (CN), are indicated at the bottom.

3 Supplemental methods

3.1 Specific mixed effects model architectures

3.1.1 Dense feedforward neural network (DFNN)

Our base DFNN architecture for both the spiral classification and the MCI conversion prediction problems contained 3 hidden dense layers with 4 neurons each (Fig. S1, blue area). Each was followed by a ReLU activation function. For binary classification, the output layer was a single-neuron dense layer with a sigmoid activation. To construct the ARMED-DFNN, we began by introducing an adversarial classifier containing 3 hidden dense layers with 8, 8, and 4 neurons, respectively (conceptualized in Fig. S1, gray area). ReLU activations were used for the hidden layers, and the output layer was a softmax layer for multi-class classification. The random effects subnetwork (Fig. S1, orange area) of the ARMED-DFNN varied by problem. For the *spiral classification* problem where the random effect was nonlinear by design, we included an 4-dimensional cluster-specific nonlinear slope (main text Eq. 4), which was multiplied with the output of the last hidden dense layer in the main (fixed effects) subnetwork, and a cluster-specific intercept (main text Eq. 5):

$$h_R(\boldsymbol{x}_i; U(\boldsymbol{z}_i)) = h_{R,nlin}(\boldsymbol{x}_i; u_{nlin}(\boldsymbol{z}_i)) + h_{R,int}(u_{int}(\boldsymbol{z}_i)) \tag{1}$$

For the *MCI conversion prediction* problem, we included a p-dimensional cluster-specific linear slope (main text Eq. 6), which was multiplied by the input X with p features, and a cluster-specific intercept (main text Eq. 5):

$$h_R(\boldsymbol{x}_i; U(\boldsymbol{z}_i)) = h_{R,lin}(\boldsymbol{x}_i; u_{lin}(\boldsymbol{z}_i)) + h_{R,int}(u_{int}(\boldsymbol{z}_i)) \tag{2}$$

To combine the fixed ($h_F(\boldsymbol{x}_i; \beta)$) and random effects ($h_R(\boldsymbol{x}_i; U(\boldsymbol{z}_i))$) subnetwork outputs and obtained the mixed effects prediction $\hat{y}_{M,i}$, we used the following additive mixing function:

$$\hat{y}_{M,i} = m\left(h_F(\boldsymbol{x}_i; \beta), h_R(\boldsymbol{x}_i; U(\boldsymbol{z}_i))\right) = sigmoid\left(h_F(\boldsymbol{x}_i; \beta)^\top \beta_L + h_R(\boldsymbol{x}_i; U(\boldsymbol{z}_i))\right) \tag{3}$$

where β_L contains the weights of the output layer in the fixed effects subnetwork. The final training objective for the ARMED-DFNN is

$$\mathcal{L}_{BCE}(\boldsymbol{y}, \hat{\boldsymbol{y}}_M) + \lambda_F \mathcal{L}_{BCE}(\boldsymbol{y}, \hat{\boldsymbol{y}}_F) + \lambda_K D_{\mathrm{KL}}(q(U)||p(U)) - \lambda_g \mathcal{L}_{CCE}(Z, \hat{Z}) \tag{4}$$

where $\mathcal{L}_{BCE}$ is the binary crossentropy loss:

$$\mathcal{L}_{BCE}(\boldsymbol{y}, \hat{\boldsymbol{y}}) = -\frac{1}{n}\sum_{i=1}^{n} y_i \log(\hat{y}_i) + (1 - y_i)\log(1 - \hat{y}_i)$$

The Z-predictor model, which infers the cluster membership matrix Z of unseen sites, used the same architecture as the adversarial classifier. All DFNN models were trained with the Adam optimizer with a learning rate of 0.001 for 50 epochs, with early stopping based on validation loss.

3.1.2 Convolutional neural network (CNN)

The base CNN used for AD vs. CN classification contained 7 blocks, each comprising a two-dimensional convolutional layer with 3×3 kernels, batch normalization, and a PReLU activation function (Fig. S3, blue area). A 2×2 max-pooling layer was used between each block. The output of the last convolutional block was flattened and fed into a 512-neuron dense layer. Here, $h_F(X; \beta)$ is defined as the output of this hidden dense layer. The output layer was a single-neuron dense layer with a sigmoid activation. To form the fixed effects subnetwork of the ARMED-CNN, we first added an adversarial classifier (Fig. S3, gray area). We used an architecture similar to the base CNN, but the output layer was replaced by a softmax layer for multi-class classification. Intermediate outputs from each layer in the original CNN were sent into the adversarial classifier at the layer with the corresponding shape. Next, the random effects subnetwork (Fig. S3, orange area) consisted of a cluster-specific bias and 512-dimensional cluster-specific scalars (nonlinear slopes, akin to Eq. 1), which was multiplied with $h_F(X; \beta)$. We used the mixing function in Eq. 3 to combine the fixed and random effects subnetwork outputs. The loss function was the same as in Eq. 4. The Z-predictor model used the same architecture as the adversarial classifier and all models were trained with the Nadam optimizer with a learning rate of 0.0001 for 20 epochs.

3.1.3 Autoencoder-classifier (AEC)

We began with the autoencoder architecture used in [1] (Fig. S4, blue area), which contains an encoder to compress an image into a 56-dimensional latent representation $e_F(X;\beta)$. A decoder then reconstructs the image $\hat{X}_F$. The encoder contained 6 blocks, each with a convolutional layer with 4×4 kernels and 2×2 striding, batch normalization, and PReLU activation. The output of the last convolutional block was flattened and fed into a 56-neuron dense layer with produced the compressed latent representation. The decoder architecture was symmetric and replaced convolutional layers with transposed convolutional layers. To simultaneously perform classification, we introduced an auxiliary classifier subnetwork which predicts the cell phenotype $\hat{\boldsymbol{y}}_F$, i.e. high vs. low metastatic efficiency, from the latent representation. The auxiliary classifier took the encoder's latent representation as input and contained a 32-neuron hidden layer and a sigmoid output layer.

To create the fixed effects subnetwork, we added an adversarial classifier to predict each image's cluster $\hat{Z}_A$ from the layer activations of the encoder (Fig. S4, gray area). Through the generalization loss, the autoencoder is penalized for learning features that allow accurate cluster prediction. The encoder and decoder weights are tied so that this penalty affects both modules of the autoencoder. The adversary used the same architecture as the encoder, with the addition of a final softmax output layer.

Because image-level batch effects are believed to pervade all levels of the feature hierarchy, including lower level pixel-level features as well as higher-level morphology features, we construct a second, mirrored autoencoder as the random effects subnetwork (Fig. S4, orange area). In this second autoencoder, called the RE-AEC, random effect features are learned at every layer using random effects convolution (RECON) blocks (Fig. S5), which were inspired by style transfer networks [2, 3]. RECON blocks replaced the batch normalization layers of the original autoencoder. After a convolutional layer, these blocks apply instance normalization. In contrast to batch normalization which normalizes each mini-batch to zero mean and unit variance, instance normalization normalizes each sample independently [3]. Afterward, a cluster-specific random scale and bias is applied to each feature map, effectively rescaling and shifting the convolution outputs in a cluster-dependent manner.

The RE-AEC produces a cluster effect-laden latent representation $e_R(X;U(Z))$ and reconstruction $\hat{X}_R$. To further enforce the learning of cluster-specific features in the RE-AEC, we added two more classifiers to predict cluster membership from the latent representation, $\hat{Z}_L$, and from the image reconstruction, $\hat{Z}_I$. By minimizing the prediction error of these two classifiers, the RE-AEC is encouraged to produce latent representations and reconstructions characterizing the cluster effects.

To create the ARMED-AEC, we combined the fixed effects subnetwork (the domain adversarial AEC, denoted as DA-AEC) with the RE-AEC. Specifically, we concatenated the latent representations $e_F(X;\beta)$ and $e_R(X;U(Z))$ into a single vector and used this as input to a separate DFNN classifier trained to produce a mixed effects-based phenotype prediction $\hat{\boldsymbol{y}}_M$. In other words, the mixing function was

$$\hat{y}_{M,i} = m\left(h_F(\boldsymbol{x}_i;\beta), h_R(\boldsymbol{x}_i;U(\boldsymbol{z}_i))\right) = m([h_F(\boldsymbol{x}_i;\beta), h_R(\boldsymbol{x}_i;U(\boldsymbol{z}_i))])$$

where $m(...)$ represents this new classifier using the concatenated latent representations as input. The combined training objective of the ARMED-AEC was

$$\begin{aligned}
&\lambda_{recon,F}\mathcal{L}_{MSE}(X,\hat{X}_F) + \lambda_{recon,R}\mathcal{L}_{MSE}(X,\hat{X}_R) \\
&+ \lambda_{class,F}\mathcal{L}_{BCE}(\boldsymbol{y},\hat{\boldsymbol{y}}_F) + \lambda_{class,M}\mathcal{L}_{BCE}(\boldsymbol{y},\hat{\boldsymbol{y}}_M) \\
&+ \lambda_K D_{\mathrm{KL}}(q(U)||p(U)) \\
&+ \lambda_L\mathcal{L}_{CCE}(Z,\hat{Z}_L) + \lambda_I\mathcal{L}_{CCE}(Z,\hat{Z}_I) \\
&- \lambda_g\mathcal{L}_{CCE}(Z,\hat{Z}_A)
\end{aligned} \tag{5}$$

The first line in Eq. 5 contains the image reconstruction loss for the fixed and random effects subnetworks. Here, we use the mean squared error (MSE) between the input X and the reconstruction $\hat{X}$. The second line contains the phenotype classification loss (binary crossentropy) between the true and predicted phenotype labels $\boldsymbol{y}$ and $\hat{\boldsymbol{y}}$. The third line is the KL divergence for Bayesian layers. The fourth line contains the categorical crossentropy between the true cluster label Z and the cluster labels predicted by the latent-cluster classifier $\hat{Z}_L$ and the image-cluster classifier $\hat{Z}_I$. Finally, the last line is the cluster generalization loss

which encourages the fixed effects subnetwork to learn features that prevent the adversary from predicting cluster labels $\hat{Z}_A$.

We used an additional CNN classifier analogous to the adversarial classifier as the Z-predictor. All AEC models were trained with the Nadam optimizer with a learning rate of 0.0001 for 20 epochs, with early stopping based on reconstruction mean squared error on validation data.

3.2 Spiral classification simulations

The spiral simulations generated two spirals, labeled $y = 0$ and $y = 1$ and separated in phase by π radians (see Fig. S2 and Eq. 6). We randomly generated $n = 10,000$ points along the spirals at arc lengths between 0 and 2π. Consequently, the measured features for the ith point are its two-dimensional coordinates $x_{i,1}$ and $x_{i,2}$ and the label is y_i. We then added Gaussian noise $\eta \sim N(0, 0.1)$ to X to increase the classification challenge.

$$\begin{aligned} x_{i,1} &= -\frac{r_j t_i}{2\pi}\cos(t_i - \phi) + \eta \\ x_{i,2} &= \frac{r_j t_i}{2\pi}\sin(t_i - \phi) + \eta \\ t &\in \mathbb{R}^{n\times 1} \sim \text{Uniform}(0, 2\pi) \\ \phi &= \begin{cases} 0, & y = 0 \\ \pi, & y = 1 \end{cases} \end{aligned} \tag{6}$$

To simulate random effects, we divided the points evenly into $c = 10$ clusters and varied the spiral radius r_j for each cluster j. In simulation 1, we sampled the cluster-specific radii from a normal distribution centered at 1, $r_j \sim N(1, 0.3)$ (Fig. S2a). In simulation 2, we increased the severity of the random perturbations by sampling the cluster-specific radii from a normal distribution centered at 0, $r_j \sim N(0, 1.0)$, which causes the spirals to be inverted in half of the clusters (Fig. S2b). In simulation 3, we added two new features x_3 and x_4 which were confounded, i.e. they were associated with spiral label, y, but uncorrelated with the underlying spiral function. To simulate confounded variables, we first sampled a random variable $\rho_j \sim N(0, 0.1)$ for each cluster. We let ρ_j determine the class balance for each cluster, e.g. in cluster 3 with $\rho_3 = 0.2$, 20% of samples belong to class $y = 0$ and 80% belong to class $y = 1$. We then generated two additional data features as a function of ρ_j (Eq. 7), thereby creating a confounded relationship between x and y that is unrelated to the true underlying spiral function (Fig. S2c).

$$\begin{aligned} x_{i,3} &\sim N(\rho_j, 0.2) \\ x_{i,4} &\sim N(\rho_j, 0.2) \end{aligned} \tag{7}$$

3.3 Alzheimer's Disease and mild cognitive impairment clincal and neuroimaging datasets

Data used in the preparation of this article were obtained from the Alzheimer's Disease Neuroimaging Initiative (ADNI) database (`https://adni.loni.usc.edu`). The ADNI was launched in 2003 as a public-private partnership, led by Principal Investigator Michael W. Weiner, MD. The primary goal of ADNI has been to test whether serial magnetic resonance imaging (MRI), positron emission tomography (PET), other biological markers, and clinical and neuropsychological assessment can be combined to measure the progression of mild cognitive impairment (MCI) and early Alzheimer's disease (AD).

3.3.1 MCI conversion prediction dataset

The DFNN models for the classification of progressive vs. stable MCI (pMCI vs. sMCI) were trained on the curated "ADNIMERGE" dataset provided by ADNI. This dataset contains a selection of key variables from the ADNI study. We selected the subjects with the MCI diagnosis at the baseline visit and who had a 24-month follow-up visit. We then selected the 20 sites with the most subjects and held out the remaining 34 sites as the "unseen site" testing data. Subjects were labeled pMCI if their 24-month diagnosis changed

to Alzheimer's Disease and sMCI otherwise. The 20 "seen sites" contained 392 subjects including 106 (27%) pMCI subjects and 286 (73%) sMCI subjects. We performed 10×10 fold nested cross-validation to optimize model hyperparameters and we report test performance on the outer folds in the results. The unseen sites contained 313 subjects including 123 (38%) pMCI subjects and 190 (62%) sMCI subjects. We selected 37 features with measurements available in at least 70% of the subjects. These included:

- Demographics: gender, ethnicity, race, marital status, years of education, and age
- Cognitive scores: Clinical Dementia Rating - Sum of Boxes (CDR-SB), Alzheimer's Disease Assessment Scale - Cognitive Subscale, 11-item and 13-item versions (ADAS11, ADAS13), ADAS word recognition score, Mini Mental State Exam (MMSE), Rey Auditory Verbal Learning Test (RAVLT) subscores, Trail Making Test - Part B score, Functional Activities Questionnaire, modified Preclinical Alzheimer's Cognitive Composite (mPACC) with digit symbol substitution score and with Trails B score
- Region volumes from structural MRI: ventricles, hippocampus, whole brain, entorhinal cortex, fusiform gyrus, medial temporal lobe, and intracranial volume
- Fluorodeoxyglucose PET: average of the mean regional standardized uptake values (SUVr) from each of the following regions: angular gyrus, temporal lobe, and posterior cingulate gyrus
- Cerebrospinal fluid concentrations: tau, phosphorylated tau, beta-amyloid
- Number of APOE4 alleles

Addition of confounded probes to the ADNI data We simulated 5 confounded probe features to test each model's ability to discern confounding effects which are spuriously correlated with the prediction target. First, we computed the percentage of pMCI subjects ρ_j for each site j. We then generated the following 5 features as linear and nonlinear functions of ρ_j:

$$x_1 = \rho_j + \eta$$
$$x_2 = \rho_j^2 + \eta$$
$$x_3 = \frac{1}{\rho_j} + \eta$$
$$x_4 = \cos \rho_j + \eta$$
$$x_5 = \sin \rho_j + \eta$$

where η is Gaussian noise with $\eta \sim N(0, 0.05)$. Consequently, these probe features were associated with the prediction target, MCI conversion, without having any biological relevance. In the experiments, we evaluate the sensitivity of each model to these probes.

3.3.2 AD diagnosis dataset

The data for training CNN classifiers of AD vs. cognitively normal (CN) individuals was selected from the ADNI2 and ADNI3 cohorts. To emphasize the underlying confounding site effect in this dataset, we selected 12 study sites that differed in scanner manufacturer, scanner model, and the proportion of AD samples (Table S1). This included 5 sites using General Electric scanners with a high proportion of AD samples ($\geq$ 50%) and 7 sites using Philips or Siemens scanners with a low proportion of AD samples ($\leq$ 11.3%). Consequently, there is a strong confounding effect due to the artifactual association between image acquisition characteristics and the likelihood of an AD diagnosis.

We obtained 2D coronal slices as follows. First, T1-weighted MRI were skullstripped using CONSNet [4]. The results were manually inspected and images with poor skullstripping were discarded. Next, a nonlinear transformation between the skullstripped images and the Montreal Neurological Institute (MNI) ICBM152 brain template was computed using ANTs [5–7]. We selected a point in the right hippocampus at MNI coordinate ($x = 32, y = -6, z = -26$). We transformed this coordinate back into subject space using the

inverse transformation from ANTs and extracted the coronal slice orthogonal to the anterior-posterior axis in subject space and passing through this coordinate. Finally, we normalized image intensity by scaling the 2nd and 98th percentiles of the intensity histogram to 0 and 255, respectively.

This resulted in a final dataset containing the 12 "seen sites" included 482 images from 184 subjects. Given this sample size, we chose to perform Monte Carlo cross-validation with 10 random splits instead of K-fold cross-validation. For each split, the subjects were divided into 70% training, 10% validation, and 20% test partitions, with all images from each subject assigned to the same partition. Model hyperparameters were optimized based on validation performance and final test performance on the 12 seen sites is reported in the results. The "unseen sites" dataset contained 1,703 images from 673 subjects, from the remaining 51 sites, which was used to evaluate the performance of the models on sites not used during training.

3.4 Melanoma live-cell image dataset

Complete details of this dataset can be found in Zaritsky et al. 2021 [1]. Here, we briefly describe the data included in the current analysis. We selected images from 7 patient-derived xenografts (PDXs) transplanted from melanoma patients to immunocompromised mice. These included 4 PDXs categorized as low metastatic efficiency, meaning the patient had metastases only to the lung and was successfully treated, and 3 PDXs categorized as high metastatic efficiency, meaning the patient had widespread metastases and died. Cells from each PDX were placed on collagen plates and recorded with phase contrast microscopy, at a rate of 1 image per minute for 2 hours, over 24 days (i.e. batches). Not every PDX was imaged in each batch, so we selected 13 batches where cells from at least 2 PDXs were imaged to constitute the "seen batches" dataset. This contained 120,863 images from 3,260 cells. We divided this dataset into 70% training, 10% validation, and 20% test partitions, with all images from the same cell assigned to the same partition. Performance on the test partition is reported in the results. The "unseen batches" dataset contained 10,699 images from 2,351 cells, imaged over the remaining 11 batches.

References

[1] A. Zaritsky, A. R. Jamieson, E. S. Welf, A. Nevarez, J. Cillay, U. Eskiocak, B. L. Cantarel, and G. Danuser, "Interpretable deep learning uncovers cellular properties in label-free live cell images that are predictive of highly metastatic melanoma," *Cell Systems*, vol. 12, no. 7, pp. 733–747.e6, 2021.

[2] V. Dumoulin, J. Shlens, and M. Kudlur, "A Learned Representation For Artistic Style," in *International Conference on Learning Representations*, 2017. [Online]. Available: https://arxiv.org/pdf/1610.07629.pdf

[3] D. Ulyanov, A. Vedaldi, and V. Lempitsky, "Improved Texture Networks: Maximizing Quality and Diversity in Feed-Forward Stylization and Texture Synthesis," in *CVPR*, 2017, pp. 6924–6932.

[4] O. Lucena, R. Souza, L. Rittner, R. Frayne, and R. Lotufo, "Convolutional neural networks for skull-stripping in brain MR imaging using silver standard masks," *Artificial Intelligence in Medicine*, vol. 98, pp. 48–58, 2019. [Online]. Available: https://pubmed.ncbi.nlm.nih.gov/31521252/

[5] B. B. Avants, N. J. Tustison, G. Song, P. A. Cook, A. Klein, and J. C. Gee, "A reproducible evaluation of ANTs similarity metric performance in brain image registration," *NeuroImage*, vol. 54, no. 3, pp. 2033–2044, 2011.

[6] N. J. Tustison, P. A. Cook, A. Klein, G. Song, S. R. Das, J. T. Duda, B. M. Kandel, N. van Strien, J. R. Stone, J. C. Gee, and B. B. Avants, "Large-scale evaluation of ants and freesurfer cortical thickness measurements," *NeuroImage*, vol. 99, pp. 166–179, 2014.

[7] V. S. Fonov, A. C. Evans, R. C. McKinstry, C. R. Almli, and D. L. Collins, "Unbiased nonlinear average age-appropriate brain templates from birth to adulthood," *NeuroImage*, vol. 47, p. S102, 2009.
